# Type 2 Diabetes Leads to Axon Initial Segment Shortening in *db/db* Mice

**DOI:** 10.3389/fncel.2018.00146

**Published:** 2018-06-08

**Authors:** Leonid M. Yermakov, Domenica E. Drouet, Ryan B. Griggs, Khalid M. Elased, Keiichiro Susuki

**Affiliations:** ^1^Department of Neuroscience, Cell Biology, and Physiology, Boonshoft School of Medicine, Wright State University, Dayton, OH, United States; ^2^Department of Pharmacology and Toxicology, Boonshoft School of Medicine, Wright State University, Dayton, OH, United States

**Keywords:** axon initial segment, medial prefrontal cortex, hippocampus, type 2 diabetes, exercise, *db/db* mice

## Abstract

Cognitive and mood impairments are common central nervous system complications of type 2 diabetes, although the neuronal mechanism(s) remains elusive. Previous studies focused mainly on neuronal inputs such as altered synaptic plasticity. Axon initial segment (AIS) is a specialized functional domain within neurons that regulates neuronal outputs. Structural changes of AIS have been implicated as a key pathophysiological event in various psychiatric and neurological disorders. Here we evaluated the structural integrity of the AIS in brains of *db/db* mice, an established animal model of type 2 diabetes associated with cognitive and mood impairments. We assessed the AIS before (5 weeks of age) and after (10 weeks) the development of type 2 diabetes, and after daily exercise treatment of diabetic condition. We found that the development of type 2 diabetes is associated with significant AIS shortening in both medial prefrontal cortex and hippocampus, as evident by immunostaining of the AIS structural protein βIV spectrin. AIS shortening occurs in the absence of altered neuronal and AIS protein levels. We found no change in nodes of Ranvier, another neuronal functional domain sharing a molecular organization similar to the AIS. This is the first study to identify AIS alteration in type 2 diabetes condition. Since AIS shortening is known to lower neuronal excitability, our results may provide a new avenue for understanding and treating cognitive and mood impairments in type 2 diabetes.

## Introduction

Type 2 diabetes is associated with increased risk of developing mild cognitive impairment, mood disorders, or dementia and interferes with the daily life of patients (Stoeckel et al., 2016). Studies in patients with type 2 diabetes indicate functional and neuroanatomical abnormalities in prefrontal gray matter (Kumar et al., 2008) and hippocampus (Gold et al., 2007). Detailed mechanism of how diabetes leads to these and other neurological complications is not fully understood. Previous studies investigating diabetic brain complications focused mainly on neuronal loss (Ramos-Rodriguez et al., 2013; Wang et al., 2016; Infante-Garcia et al., 2017) or altered *neuronal input* in the form of synaptic plasticity (Li et al., 2002; Stranahan et al., 2008) although normal brain function relies on both neuronal input and output signals. The impact of type 2 diabetes on *neuronal output* remains a critical gap in knowledge.

A key structure for neuronal output is the axon initial segment (AIS), a specialized functional domain within neurons that lies between the soma and the axon proper (Rasband, 2010). Synaptic inputs into the neuron are integrated and converge on the initiation of action potentials at the AIS that are propagated along axons (Bender and Trussell, 2012; Kole and Stuart, 2012). Thus, the AIS contributes to transmitting information and enabling network communication between neurons. The formation of the AIS is characterized by an accumulation of voltage-gated sodium channels, anchored to the neuronal cytoskeleton by submembranous scaffolding and cytoskeletal proteins such as ankyrinG and βIV spectrin (Griggs et al., 2017; Nelson and Jenkins, 2017). Importantly, activity-dependent changes in AIS structures (length and/or location), as detected by ankyrinG or βIV spectrin immunofluorescence, alter excitability and firing behavior of neurons, thereby modulating neuronal output (reviewed in Yamada and Kuba, 2016; Jamann et al., 2018).

Recent human studies identified that disruption of the AIS is a key pathophysiological event in various psychiatric and neurological disorders. For example, one of the most significant genetic risk loci for schizophrenia is *ANK3* (Athanasiu et al., 2010; Roussos et al., 2012), a gene that encodes the master organizer for AIS assembly, ankyrinG (Nelson and Jenkins, 2017). Compared to the post-mortem brains of healthy controls, ankyrinG signal intensity is decreased at the AIS of pyramidal neurons in the superficial cortical layer of schizophrenic patients (Cruz et al., 2009). In addition, the presence of AIS is dramatically reduced in surviving cortical tissue adjacent to microinfarcts in humans (Coban et al., 2017). Structural alterations such as decreased AIS length or AIS loss are also reported in animal models of various brain diseases, such as stroke (Schafer et al., 2009; Hinman et al., 2013), mild traumatic brain injury (Baalman et al., 2013; Greer et al., 2013; Vascak et al., 2017), Alzheimer’s disease (Marin et al., 2016), and multiple sclerosis (Hamada and Kole, 2015; Clark et al., 2016). Thus, structural changes in the AIS are emerging as a new target of axonal plasticity and/or pathology common in a wide variety of neurological and psychiatric disorders.

Here we show that, in an established type 2 diabetes model *db/db* mice (Hummel et al., 1966; Chen et al., 1996), reduced AIS length in both prefrontal cortex and hippocampus is associated with signs of diabetes. These results provide new insights into pathophysiology of neuropsychiatric complications in type 2 diabetes.

## Materials and Methods

### Animals

Male and female *db/db* (BKS.Cg-*Dock7^m^*+/+*Lepr^db^*/J; RRID:IMSR_JAX:000642) mice and their age-matched non-diabetic heterozygote littermates (hereafter referred to as controls) aged 5–11 weeks were used. Mice were housed within the Laboratory Animal Resources at Wright State University in groups of three to four per cage at 22–24°C under 12-h light/12-h dark conditions with *ad libitum* access to food and water. All animal procedures were approved by the Institutional Animal Care and Use Committee at Wright State University (Animal Use Protocol # 1113), and conform to the United States Public Health Service Policy on Humane Care and Use of Laboratory Animals.

### Measurement of Blood Glucose and Hemoglobin A1c (HbA1c)

A small incision was made on the tip of the tail to obtain a drop of blood. Blood glucose and HbA1c were measured by collecting a drop of blood onto either a TrueMetrix blood glucose test strip (Trividia Health, Fort Lauderdale, FL, USA) or the A1CNow+ blood collection cartridge (PTS Diagnostics, Indianapolis, IN, USA). Blood glucose was measured twice from the same tail incision for better accuracy. For weekly monitored blood glucose levels, the measurements were taken 2–3 h after the start of the 12-h light cycle.

### Exercise Treatment Experimental Design

At 5 weeks of age, *db/db* mice and controls were subjected to forced wheel running using an automated system to control running wheel speed and duration of rotation (Lafayette Instrument, Lafayette, IN, USA) as described previously (Somineni et al., 2014). Mice were trained to run within the wheel over a 5 days period. The initial rotation speed of the wheels was set at 4 m/min and increased daily by 1 m/min until a speed of 8 m/min was reached on d5 of training. Similarly, the duration of exercise increased daily from 15 min to 1 h in 15-min increments. After the last day of training (d5), mice were binned into exercise and no exercise groups. The exercise regimen lasted for 5 weeks from 5 weeks of age until 10–11 weeks of age. Mice in the exercise group were placed in the rotating wheels 7 days/week for 1 h/day at 8 m/min. Exercise sessions took place 2–3 h before the end of the 12-h dark cycle. Glucose and weight were monitored weekly. The last glucose and HbA1c measurements were collected at 10 weeks of age, several days before completion of exercise treatment and terminal collection of tissues for immunostaining and western blotting analyses in control and *db/db* mice aged 10–11 weeks. Mice were euthanized at the end of exercise treatment 5 h after their last exercise session.

### Immunostaining

Mice were sacrificed by isoflurane overdose and brains were immediately removed and fixed in ice-cold 4% paraformaldehyde in 0.1 M phosphate buffer for 90 min, cryoprotected overnight in 20% sucrose, blocked and placed in custom-made foil molds, frozen in TissueTek O.C.T. (4583, Sakura Finetek, Torrance, CA, USA), and stored at −80°C. Coronal brain sections (35 μm) were cut using a cryostat (HM550, Thermo Scientific, Waltham, MA, USA) at 1.7 mm ± 0.15 mm (medial prefrontal cortex), 1.1 mm ± 0.20 mm (corpus callosum) and −1.64 mm ± 0.20 mm (hippocampus) relative to bregma. Staining was performed using free floating sections with gentle rocking. Sections were blocked for 1 h in 0.1 M phosphate buffer (pH 7.4) containing 0.3% Triton X-100 and 10% goat serum (PBTGS), then incubated overnight at 4°C with primary antibodies diluted in PBTGS. Samples were washed three times for 10 min in PBTGS, followed by incubation in the dark with fluorescently labeled secondary antibodies for 1 h at room temperature. To detect cell nuclei, sections were counterstained with Hoechst 33342 (Cat# H3570, Thermo Fisher Scientific, IL, USA). Finally, immunolabeled sections were washed once in PBTGS, and twice in 0.1 M phosphate buffer containing 0.15% Triton X-100, then carefully mounted onto slides using mounting medium (Cat# 71-00-16, KPL, Gaithersburg, MD, USA).

### Antibodies

The following primary antibodies were used: mouse monoclonal ankyrinG (N106/36, UC Davis/NIH NeuroMab Facility Cat# 75-146 RRID:AB_10673030), NeuN (Millipore Cat# MAB377 RRID:AB_2298772), myelin basic protein (MBP; BioLegend Cat# 808401, RRID:AB_2564741), GAPDH (Enzo Life Sciences Cat# ADI-CSA-335-E, RRID:AB_2039148); rabbit polyclonal Caspr (Abcam Cat# ab34151 RRID:AB_869934), βIV spectrin (M.N. Rasband, Baylor College of Medicine; TX, USA; Cat# βIV SD RRID:AB_2315634), Kv1.2 (Zhang et al., 2013), cleaved caspase 3 (Cell Signaling Technology Cat# 9661, RRID:AB_2341188); chicken polyclonal neurofascin (NF; R&D Systems Cat# AF3235 RRID:AB_10890736). Alexa Fluor (594, 488, 350) or AMCA conjugated secondary antibodies were used for immunohistochemistry and peroxidase conjugated anti-mouse, -rabbit, or -chicken secondary antibodies for western blotting (Jackson ImmunoResearch Laboratories, West Grove, PA, USA).

### Image Capture and AIS Measurements

All images were captured on an Axio Observer Z1 with Apotome 2 fitted with a Axiocam Mrm CCD camera (ZEISS, Thornwood, NY, USA). For AIS analysis, three-dimensional z-stack images were taken of the prelimbic area of the medial prefrontal cortex at layers II/III (2015 Allen Institute for Brain Science. Allen Brain Atlas. Available from: mouse.brain-map.org. Reference atlas, coronal atlas, image 39 of 132) and pyramidal layer of CA1 of the hippocampus (Allen Brain Atlas, image 72 of 132). In medial prefrontal cortex, AIS measurements were taken from the full mix of glutamatergic and γ-aminobutyric acid (GABA)-ergic neurons in this region, of which about every one out of five neurons is GABAergic (Hendry et al., 1987; Sahara et al., 2012). In hippocampus, AIS length was measured in large neurons, most of them are presumably pyramidal cells. Z-stacks were then loaded in Fiji (Schindelin et al., 2012). Image black and white values of βIV spectrin immunostaining were inverted to display black AIS on white background. AIS were defined as βIV spectrin-labeled segments greater than 10 μm in length with clearly identifiable start and end points. AIS start point was defined as sharply increased βIV spectrin signal closest to the soma. AIS end point was defined as reduced βIV spectrin signal to the point where it could no longer be discerned from the background. AIS that had blunt ends were excluded, as this is likely an artifact of cutting through the AIS during sectioning. The AIS length measurement was performed by carefully tracing the shape of the AIS using segmented line tool in Fiji. The length was measured in around 20 AIS per image, and three images were analyzed per mouse. Measurement of distance from soma to AIS start point was performed as described previously (Harty et al., 2013). In brief, the shortest possible distance between the neuronal soma, identified by NeuN staining, and the most proximal portion of the AIS, identified by βIV spectrin staining, was measured with the straight-line tool in Fiji. Measurement of AIS density was performed per field of view (223.82 × 167.70 μm) in most superficial part of layers II/III of the cortex (3 fields of view per animal). For node of Ranvier analysis, nodal gap or paranodal length within the corpus callosum were measured using ZEN 2.3 software (Zeiss). Image quantifications were performed by observers blinded to the identity of the images.

### Western Blotting

Mice were sacrificed by isoflurane overdose and brains were immediately removed. Frontal lobe and hippocampus were dissected, and flash frozen in liquid nitrogen and stored at −80°C until processing. Brain tissues were homogenized in ice-cold RIPA buffer (25 mM Tris HCl at pH 7.5, 150 mM NaCl, 1% Triton x100, 0.5% Deoxycholate, 0.1% SDS, 10 mM EDTA) using a pestle (BioMasher, Takara Bio, USA). Homogenates were centrifuged at 12,000× *g* for 10 min at 4°C in a Sorvall Legend Micro 21R centrifuge (Thermo Scientific). After centrifugation, supernatant was collected into fresh, ice-cold tubes and protein concentrations were measured using a Pierce BCA Protein Assay (Thermo Scientific, Cat# 23225). Samples (10 μg protein) were denatured at 95°C for 5 min in reducing sample buffer (Bio-Rad, Cat# 1610710 and #1610747), then run on a 4%–20% Mini-PROTEAN TGX stain-free gel (Bio-Rad, Cat# 4568096). The gel was then transferred to nitrocellulose membrane, 0.45 μm pore size (Bio-Rad, Cat# 1620115). Membranes were blocked with 20 mM Tris, pH 8.0 and 0.05% (v/v) Tween 20 (TBST) containing 4% (w/v) milk for 1 h. Primary antibodies were diluted in TBST with milk at 1:1000, added to the membranes and incubated overnight at 4°C. Primary antibody was washed and horseradish peroxidase (HRP) conjugated secondary antibodies (1:10,000) were incubated for 1 h at room temperature. Signals generated by Pierce ECL Plus Western Blotting Substrate (Thermo Scientific, Cat# 32132) were detected using a ChemiDoc MP Imaging System (Bio-Rad). Quantification of the band density was performed using ImageLab software from Bio-Rad. The densities of the bands of interest were normalized to the relative expression of GAPDH. Total protein staining was used to confirm appropriate protein loading and transfer.

### Statistical Analyses

Comparison of the means between two groups was performed using unpaired, Student’s *t*-test. Comparison between multiple groups was performed via a two-way ANOVA followed by Tukey’s multiple comparisons tests. An alpha value of α = 0.05 was used to determine statistical significance. Correlation between AIS length and levels of blood glucose or HbA1c were analyzed by calculating the Pearson’s correlation coefficient. All data were analyzed using Prism 7.0 (GraphPad, La Jolla, CA, USA) and presented as scatter plots with mean ± SEM. Data within Results text is reported as mean ± SEM unless otherwise noted.

## Results

### AIS Length Is Reduced in *db/db* Mouse Prefrontal Cortex

**Figure 1:**
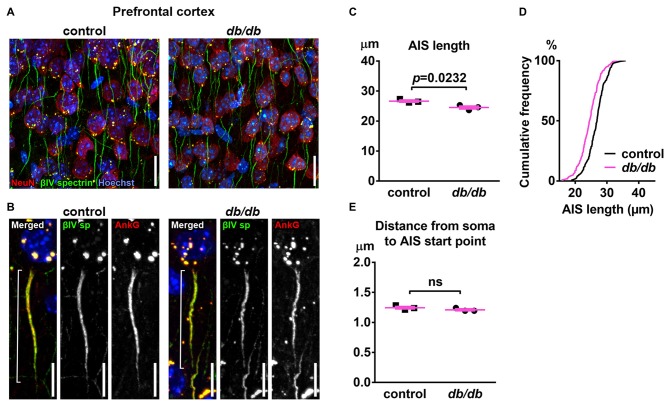
Type 2 diabetic *db/db* mice have shortened axon initial segment (AIS) in medial prefrontal cortex. **(A)** Representative images depicting the medial prefrontal cortex, prelimbic area, in 10-week-old female control (left) and *db/db* (right) mice. Brain sections were labeled for βIV spectrin (green, AIS), NeuN (red, neuronal soma) and Hoechst (blue, cell nuclei). Scale bars = 20 μm. **(B)** High magnification images of a single neuron and its AIS, labeled for βIV spectrin (green, AIS), ankyrinG (red, AIS) and Hoechst (blue, cell nuclei). Brackets show AIS length. Scale bars = 10 μm. **(C)** AIS length in medial prefrontal cortex of 10-week-old female *db/db* mice was shorter than controls (*n* = 3 mice in each group). **(D)** Cumulative frequency distribution plot of AIS lengths in medial prefrontal cortex shows a leftward shift with no change in shape in *db/db* mice compared to controls. Control, total 194 AISs from three mice; *db/db*, total 157 AISs from three mice. **(E)** Distance from soma to AIS start point in medial prefrontal cortex of 10-week-old female control and *db/db* mice was similar (*n* = 3 mice in each group). ns = not significant.

To test the hypothesis that type 2 diabetes leads to structural alteration of the AIS, we first analyzed the brains of female *db/db* mice at 10 weeks of age. These *db/db* mice had profoundly elevated blood glucose levels (529.8 ± 32.3 mg/dL, *n* = 3 mice) compared to controls (138.5 ± 2.5 mg/dL, *n* = 3 mice; *p* = 0.0003, unpaired *t*-test), as previously reported (Hummel et al., 1966). To visualize AIS structures, we immunostained coronal brain sections with antibodies to the AIS proteins ankyrinG or βIV spectrin. In medial prefrontal cortex of both control and *db/db* mice, overall structures of neurons and AIS were mostly preserved (Figures 1A,B). However, the length of the AIS was significantly decreased in *db/db* (24.52 ± 0.48 μm, *n* = 3 mice) compared to control (26.65 ± 0.35 μm, *n* = 3 mice) mice (*p* = 0.0232, unpaired *t*-test; Figure 1C). The cumulative frequency plot of individual AIS lengths in *db/db* mice showed a leftward shift and a distribution similar to control mice (Figure 1D). We also checked for altered location of the start of the AIS relative to the soma, as both proximal and distal shifts of the AIS are recognized as mechanisms of axonal functional plasticity (reviewed in Yamada and Kuba, 2016; Jamann et al., 2018). There was no difference in distance from soma to AIS start point between control (1.245 ± 0.0207 μm, *n* = 3 mice) and *db/db* (1.209 ± 0.0158 μm, *n* = 3 mice) mice (*p* = 0.2466, unpaired *t*-test; Figure 1E). These results demonstrate that AIS shortening, but not AIS relocation, occurs in the medial prefrontal cortex of type 2 diabetic *db/db* mice.

### Development of Signs of Type 2 Diabetes in *db/db* Mice Is Associated With AIS Shortening in the Prefrontal Cortex

To determine if AIS shortening is associated with the development of type 2 diabetes in *db/db* mice, we analyzed metabolic signs of type 2 diabetes as well as the AIS in control and *db/db* mice using two complementary experiments: (i) time course: beginning of diabetes (5 weeks of age) and during fully developed diabetes (10 weeks of age); and (ii) after anti-diabetic treatment (exercise) from 5 to 10 weeks of age, the period during which elevated blood glucose and HbA1c develop. We utilized male mice for these experiments to assess potential sex differences as previously reported in *db/db* brain (Vannucci et al., 2001), and because previous reports showed altered neurobehavioral outcomes and brain physiology in male *db/db* mice at an early age (Li et al., 2002; Sharma et al., 2010; Dinel et al., 2011).

**Figure 2:**
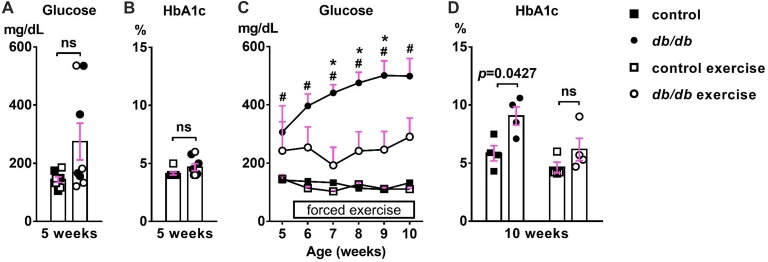
Development of elevated blood glucose and HbA1c levels and the effects of exercise treatment in *db/db* mice. **(A)** Blood glucose levels were similar in 5-week-old male control and *db/db* mice (*n* = 8). Open squares and circles represent mice binned to the exercise group, as shown in panels **(C,D)**. **(B)** HbA1c levels were similar in 5-week-old control and *db/db* mice (*n* = 8). Open squares and circles represent mice binned to the exercise group, as shown in panels **(C,D)**. **(C)** Blood glucose levels during daily exercise treatment. Exercise attenuated the development of hyperglycemia in *db/db* mice. ^#^*p* < 0.05 for control vs. *db/db*. *p < 0.05 for *db/db* vs. *db/db* exercise. **(D)** HbA1c levels at the end of exercise treatment. Two-way ANOVA (diabetes × exercise: *F*_(1,12)_ = 1.245; *p* = 0.286) followed by Tukey’s multiple comparisons test yielded the following *p* values: control vs. *db/db* (*p* = 0.0427), control vs. exercise control (*p* = 0.6548), control vs. exercise *db/db* (*p* = 0.9891), *db/db* vs. exercise control (*p* = 0.0055), *db/db* vs. exercise *db/db* (*p* = 0.0728), exercise control vs. exercise *db/db* (*p* = 0.4767).

First, we confirmed the effect of exercise on the temporal development of signs of type 2 diabetes. At 5 weeks of age, there was no significant difference in blood glucose (Figure 2A) or HbA1c (Figure 2B) levels between male control and *db/db* mice. As expected based on previous studies (Hummel et al., 1966; Sharma et al., 2010), these data suggest that 5 week old *db/db* mice are at the beginning of diabetes, since HbA1c reflects average blood glucose concentration from approximately the previous 40 days—the average red blood cell lifespan in mice (Wang et al., 2010). At 10 weeks of age, *db/db* mice had profoundly elevated blood glucose (498.875 ± 60.8 mg/dL, *n* = 4 mice) compared to controls (132.625 ± 5.832 mg/dL, *n* = 4 mice; *p* = 0.0004, two-way ANOVA followed by Tukey’s; Figure 2C). In addition, HbA1c levels were significantly elevated in *db/db* mice (9.050 ± 0.786%, *n* = 4 mice) compared to controls (5.850 ± 0.655%, *n* = 4 mice; *p* = 0.0427, two-way ANOVA followed by Tukey’s; Figure 2D). To attenuate the progression of diabetes, control and *db/db* mice aged 5 weeks were subjected to an exercise regimen using automated running wheel system (Somineni et al., 2014). Exercise treatment in *db/db* mice reduced the temporal development of elevated blood glucose (main effect of exercise in *db/db* mice; *F*_(1,6)_ = 8.641, *p* = 0.026); two-way ANOVA; Figure 2C). HbA1c levels in exercised *db/db* mice (6.175 ± 0.964%, *n* = 4 mice) were similar to that in exercised controls (4.625 ± 0.459%, *n* = 4 mice; *p* = 0.4767, two-way ANOVA followed by Tukey’s; Figure 2D). Neither age or exercise effected blood glucose or HbA1c levels in control mice. After exercise treatment at 10 weeks of age, body weight of the exercised *db/db* mice (42.875 ± 1.102 g, *n* = 4 mice) was similar to that of non-exercised *db/db* mice (46.15 ± 1.341 g, *n* = 4 mice; *p* = 0.1624, two-way ANOVA followed by Tukey’s). This is consistent with previous studies that showed beneficial effects of exercise on diabetes and its complications without body weight changes, including improved glycemic control and albuminuria (Somineni et al., 2014), reduced allodynia (Cooper et al., 2017), increased hippocampal dendritic spine density (Stranahan et al., 2009), and prevention of type 2 diabetes in patients with impaired glucose tolerance (Pan et al., 1997). Together, these data demonstrate that exercise treatment attenuated hyperglycemia, providing the appropriate experimental approach to test if the development of type 2 diabetes changes AIS morphology.

**Figure 3:**
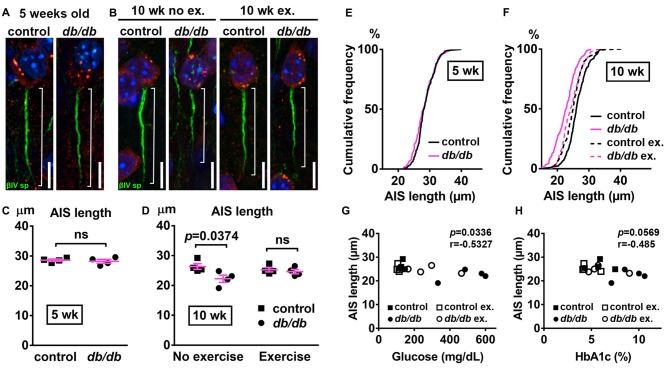
Diabetes-related AIS shortening in *db/db* mouse prefrontal cortex. **(A,B)** Representative high magnification images of a single neuron and its AIS from medial prefrontal cortex of male control (left) and *db/db* (right) mice at 5 weeks of age **(A)** and of male control and *db/db* mice with and without exercise treatment at 10 weeks of age **(B)**. Brain sections were labeled for βIV spectrin (green, AIS), NeuN (red, neuronal soma) and Hoechst (blue, cell nuclei). Brackets show AIS length. Scale bars = 10 μm. **(C)** AIS length in medial prefrontal cortex of 5-week old male control and *db/db* mice was similar (*n* = 4 mice in each group). **(D)** AIS length in medial prefrontal cortex of control and *db/db* mice with and without exercise treatment (*n* = 4 mice in each group). Two-way ANOVA (diabetes × exercise: *F*_(1,12)_ = 3.535; *p* = 0.0846) followed by Tukey’s multiple comparisons test yielded the following *p* values: control vs. *db/db* (*p* = 0.0374), control vs. exercise control (*p* = 0.8338), control vs. exercise *db/db* (*p* = 0.5686), *db/db* vs. exercise control (*p* = 0.1537), *db/db* vs. exercise *db/db* (*p* = 0.3124), exercise control vs. exercise *db/db* (*p* = 0.9625). **(E)** Cumulative frequency distribution plot of AIS lengths in medial prefrontal cortex shows similar distribution in control and *db/db* mice at 5 weeks of age. Control, total 206 AISs from four mice; *db/db*, total 193 AISs from four mice. **(F)** Cumulative frequency distribution plots of AIS lengths in medial prefrontal cortex of control and *db/db* mice at 10 weeks of age. Control without exercise, total 255 AISs from four mice; *db/db* mice without exercise, total 205 AISs from four mice; control with exercise, total 213 AISs from four mice; *db/db* mice with exercise, total 239 AISs from four mice. These four groups show similar pattern of distribution of AIS lengths, although the plot of *db/db* mice without exercise is shifted to the left. **(G,H)** Scatter plots of mean AIS lengths (y-axis) in relation to levels of blood glucose **(G)** or HbA1c **(H)** (x-axis) of individual mouse at 10 weeks of age.

Next, we examined AIS structures by immunostaining brain sections as described above. A potential confound of using *db/db* mice is that AIS shortening could be a byproduct of the *db/db* genetic model, and not the development of diabetes, as they are leptin receptor-deficient (Chen et al., 1996) and leptin signaling is involved in the neuronal and glial development of mouse embryos (Udagawa et al., 2006). However, at 5 weeks of age we observed no difference in AIS length between control (28.58 ± 0.36 μm, *n* = 4 mice) and *db/db* (28.21 ± 0.65 μm, *n* = 4 mice) mice (*p* = 0.6280, unpaired *t*-test; Figures 3A,C,E), suggesting that lack of leptin signaling does not impact AIS formation in *db/db* mice before overt signs of diabetes develop. At 10 weeks of age, in the no exercise group, AIS length was significantly shorter in male *db/db* mice (22.25 ± 1.20 μm, *n* = 4 mice) compared to controls (26.35 ± 0.97 μm, *n* = 4 mice; *p* = 0.0374, two-way ANOVA followed by Tukey’s; Figures 3B,D,F), consistent with AIS shortening in 10-week-old female *db/db* mice (Figure 1). After demonstrating that AIS shortening develops between the ages of 5–10 weeks in male *db/db* mice, we checked if AIS shortening is diminished by exercise treatment. Although the difference in AIS length between the exercising and non-exercising *db/db* mice did not reach statistical significance (*p* = 0.3124), AIS length in exercised *db/db* mice (24.63 ± 0.73 μm, *n* = 4 mice) was similar to that in exercised controls (25.25 ± 0.71 μm, *n* = 4 mice; *p* = 0.9625, two-way ANOVA followed by Tukey’s; Figures 3D,F). These results suggest that AIS shortening occurs during the period of development of type 2 diabetes from 5 to 10 weeks of age, whereas AIS shortening does not occur if hyperglycemia is controlled. Furthermore, we observed inverse correlation between AIS length and blood glucose (*r* = −0.5327, *p* = 0.0336, *n* = 16; Figure 3G) or HbA1c levels (*r* = −0.485, *p* = 0.0569, *n* = 16; Figure 3H) in these mice. Taken together, these data strongly support the idea that AIS shortening is associated with development of type 2 diabetes.

### Neuronal, Myelin, or AIS Protein Levels Are Unchanged in *db/db* Mice

**Figure 4:**
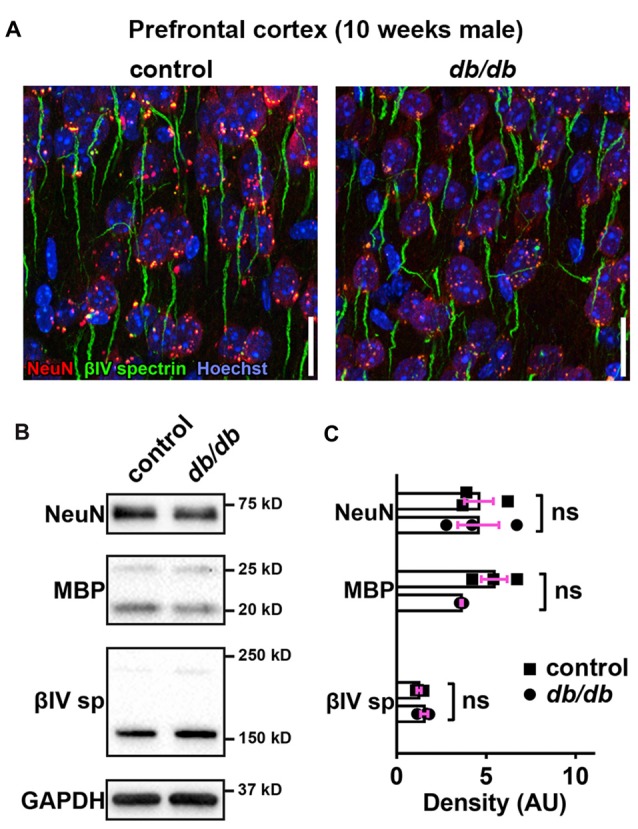
No major changes of protein levels in neurons, myelin and AIS in type 2 diabetic *db/db* mouse prefrontal cortex. **(A)** Representative images depicting the medial prefrontal cortex, prelimbic area, in 10-week-old male control (left) and *db/db* (right) mice. Brain sections were labeled for βIV spectrin (green, AIS), NeuN (red, neuronal soma) and Hoechst (blue, cell nuclei). Scale bars = 20 μm. **(B)** Representative immunoblots of homogenized frontal brain from control and *db/db* mice at 10 weeks of age. βIVsp = βIV spectrin. Uncropped images of the entire original immunoblots are shown in Supplementary Figure S2. **(C)** Quantification of relative protein band densities, normalized to GAPDH, and reported in arbitrary units (AU). No difference in protein levels was evident between control and *db/db* mice (*n* = 3). Student’s *t*-tests yielded the following *p* values: NeuN (*p* = 0.9824), both myelin basic protein (MBP) upper and lower bands (*p* = 0.0652), βIV spectrin lower band, βIV∑6 spectrin, a major isoform in adult brain (Yoshimura et al., 2017) (*p* = 0.2884).

Progressive cortical atrophy has been reported in *db/db* mice starting at 14 weeks of age (Infante-Garcia et al., 2017). To determine if reductions in neurons, myelin, or AIS protein levels could explain AIS shortening, we examined neuronal, myelin, and AIS markers in 10-week-old male control and *db/db* mice (both without exercise) using immunostaining of the prefrontal cortex and western blotting of frontal brain homogenates. The morphology in the prefrontal cortex appeared similar in control and *db/db* mice (Figures 1A, 4A). The AIS density was similar between control (32 ± 1 AIS/field of view, *n* = 4 mice) and *db/db* (33 ± 2 AIS/field of view, *n* = 4 mice) mice (*p* = 0.6471, unpaired *t*-test). Representative immunoblots are shown for NeuN, MBP, βIV spectrin and the loading control protein GAPDH (Figure 4B). Consistent with preserved cortical morphology (Figures 1A, 4A) and AIS density, there was no difference in NeuN or MBP protein level (Figure 4C). The immunoblots showed lack of cleaved caspase 3, a marker for apoptotic cell death, in both control and *db/db* brains (Supplementary Figures S1A, S4). Therefore, it is unlikely that the AIS shortening observed in 10-week-old *db/db* mice is due to loss of neurons or myelinated axons. We also found no difference in the protein level of βIV spectrin in the prefrontal cortex (Figure 4C). Together these findings suggest that the AIS shortening we observed in prefrontal cortex in diabetic animals is not secondary to gross changes in neurons, myelination, or the AIS protein expression.

### Nodal Units in Myelinated Axons of Type 2 Diabetic *db/db* Mice Are Preserved

**Figure 5:**
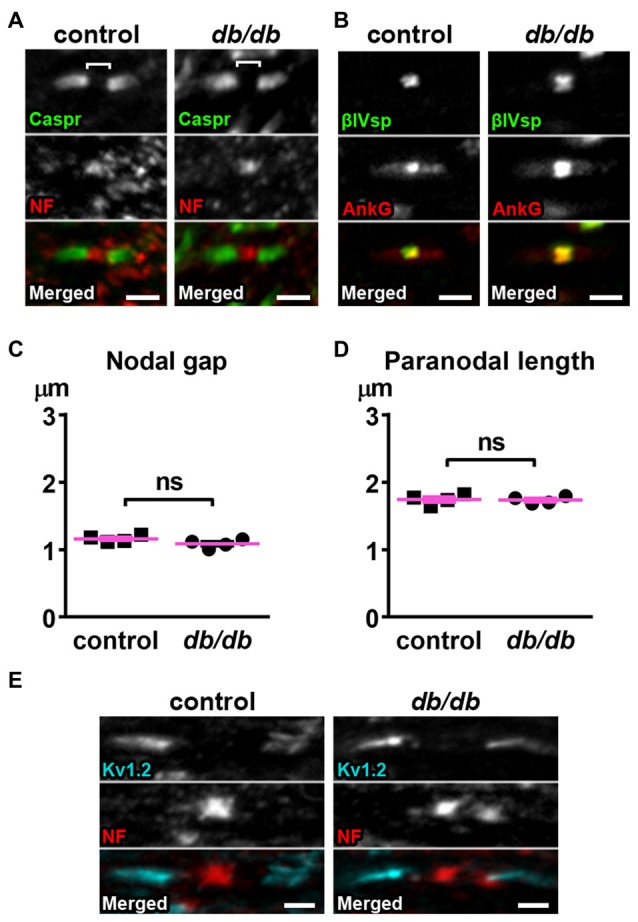
Nodes of Ranvier are preserved in corpus callosum of type 2 diabetic *db/db* mice. **(A)** Representative high magnification images of nodal units in corpus callosum of male control (left) and *db/db* (right) mice at 10 weeks of age. Caspr (green) labels paranodal junction and neurofascin (NF; red) labels the node. Brackets represent the nodal gap. Scale bars = 2 μm. **(B)** Representative high magnification images of nodal units in corpus callosum of male control (left) and *db/db* (right) mice at 10 weeks of age. βIV spectrin (βIVsp, green) labels the node and ankyrinG (AnkG, red) labels the node and paranodes. Scale bars = 2 μm. **(C)** The nodal gap, or the distance between two opposing Caspr clusters within a single nodal unit, was similar in control and *db/db* mice (*n* = 4). **(D)** The length of a single paranodal Caspr cluster was similar in control and *db/db* mice (*n* = 4). **(E)** Representative high magnification images of nodes and juxtaparanodes in corpus callosum of male control (left) and *db/db* (right) mice at 10 weeks of age. Kv1.2 (blue) labeling of the potassium channels at the juxtaparanode appeared similar in control and *db/db* mice. Scale bars = 2 μm.

The nodal unit, consisting of a node of Ranvier, paranodes and juxtaparanodes, is a critical functional domain in myelinated axons that is needed for proper nerve conduction and network communication. Importantly, the molecular organization at the node of Ranvier is nearly identical to that at the AIS, including sodium channels, NF186, ankyrinG and βIV spectrin (Griggs et al., 2017; Nelson and Jenkins, 2017). Furthermore, disruption of nodes of Ranvier has been reported in central nervous system (CNS) diseases, such as schizophrenia (reviewed in Roussos and Haroutunian, 2014) or lacunar stroke (Hinman et al., 2015), similar to AIS alterations in these same diseases. Therefore, we examined if nodal units are affected in type 2 diabetic *db/db* mouse brain at 10 weeks of age. We utilized the corpus callosum because it is a large bundle of node of Ranvier-containing white matter fibers that connects the hemispheres of the brain including the medial prefrontal cortex, and a previous study suggested that nodal alterations in corpus callosum are closely associated with onset of major depressive disorder (Miyata et al., 2016). We visualized the nodal unit using established markers of nodes of Ranvier, paranodes, and juxtaparanodes. Immunostaining of NF186 at nodes and Caspr at paranodes, the region flanking both sides of the nodes, appeared similar in male control and *db/db* mice without exercise (Figure 5A). Similarly, immunostaining of nodal ankyrinG and βIV spectrin also appeared normal (Figure 5B). The nodal gap, or the distance between two opposing Caspr clusters within a single nodal unit, was similar in control and *db/db* mice (Figure 5C). The length of a single paranodal Caspr cluster was also similar in control and *db/db* mice (Figure 5D). We also examined clusters of voltage-gated potassium channels (Kv1.2) at juxtaparanodes, since juxtaparanodal Kv1.2 was reduced in peripheral nerves in *db/db* mice and in nerve biopsies from patients with type 2 diabetes (Zenker et al., 2012). The immunostaining of Kv1.2 channel clusters at juxtaparanodes within the corpus callosum appeared similar in control and *db/db* mice (Figure 5E). These results demonstrate that, despite significant AIS shortening in medial prefrontal cortex (Figures 1, 3), the structures comprising nodal units within the corpus callosum of type 2 diabetic *db/db* mice appear normal. This is consistent with similar protein levels of NeuN and MBP in control and *db/db* mouse prefrontal cortex (Figures 4B,C).

### AIS Is Shortened in the Hippocampus of Type 2 Diabetic *db/db* Mice

**Figure 6:**
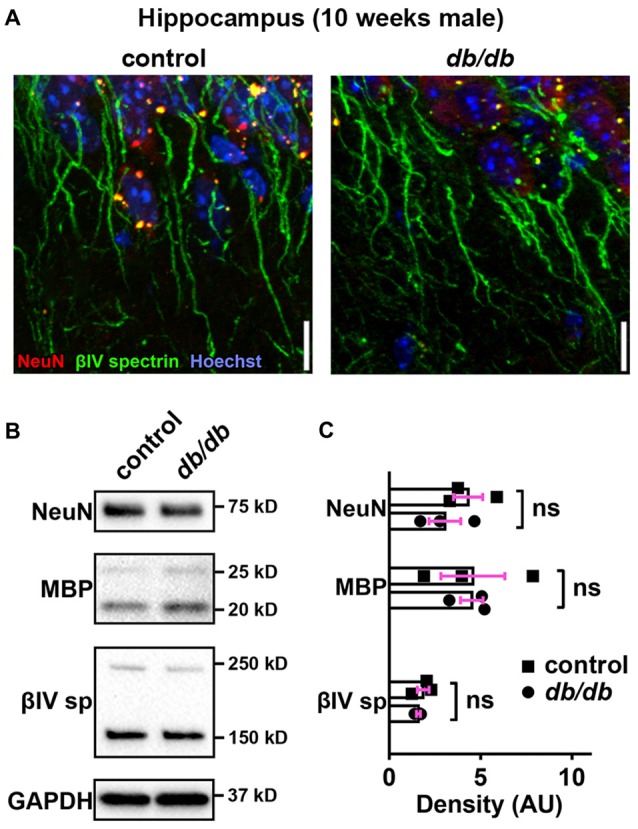
No major changes of protein levels in neurons, myelin and AIS in type 2 diabetic *db/db* mouse hippocampus. **(A)** Representative images depicting hippocampus, CA1 area, in 10-week-old male control (left) and *db/db* (right) mice (both without exercise treatment). Brain sections were labeled for βIV spectrin (green, AIS), NeuN (red, neuronal soma) and Hoechst (blue, cell nuclei). Scale bars = 10 μm. **(B)** Representative immunoblots of homogenized hippocampus from control and *db/db* mice at 10 weeks of age (both with no exercise). βIVsp = βIV spectrin. Uncropped images of the entire original immunoblots are shown in Supplementary Figure S3. **(C)** Quantification of relative protein band densities, normalized to GAPDH, and reported in arbitrary units (AU). No difference in protein levels was evident between control and *db/db* mice (*n* = 3). Student’s *t*-tests yielded the following *p* values: NeuN (*p* = 0.3401), both MBP upper and lower bands (*p* = 0.9782), βIV spectrin lower band, βIV∑6 spectrin, a major isoform in adult brain (Yoshimura et al., 2017) (*p* = 0.4822).

**Figure 7:**
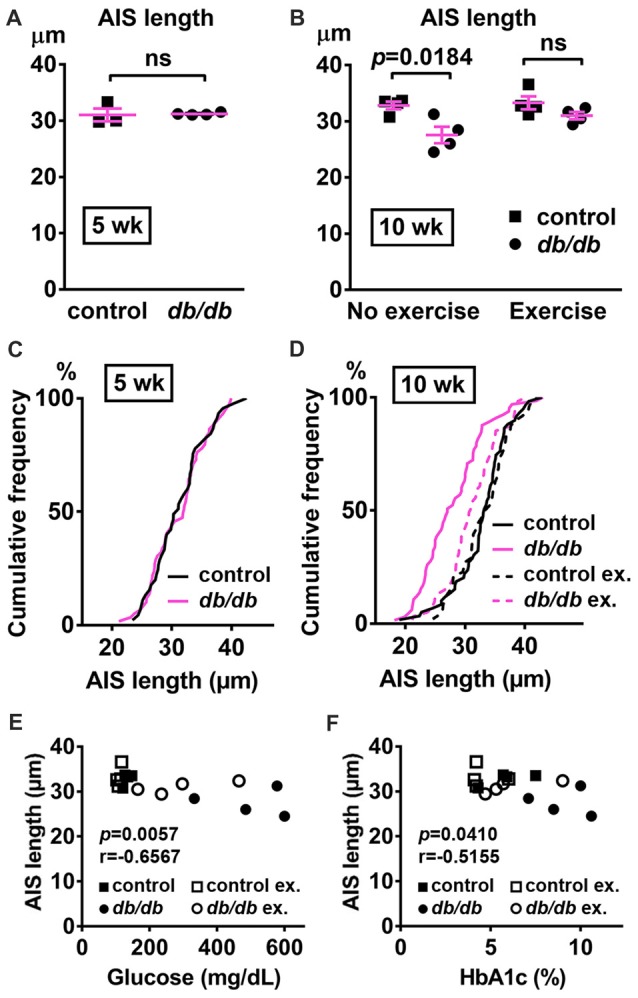
AIS is shortened in the hippocampus of type 2 diabetic *db/db* mice. **(A)** AIS length in hippocampus of 5-week old male control and *db/db* mice was similar (*n* = 3 mice in control group; *n* = 4 in *db/db* group). **(B)** AIS length in hippocampus of control and *db/db* mice with and without exercise treatment (*n* = 4 mice in each group). Two-way ANOVA (diabetes × exercise: *F*_(1,12)_ = 2.002; *p* = 0.1825) followed by Tukey’s multiple comparisons test yielded the following *p* values: control vs. *db/db* (*p* = 0.0184), control vs. exercise control (*p* = 0.9871), control vs. exercise *db/db* (*p* = 0.6311), *db/db* vs. exercise control (*p* = 0.0103), *db/db* vs. exercise *db/db* (*p* = 0.1454), exercise control vs. exercise *db/db* (*p* = 0.4449).** (C)** Cumulative frequency distribution plot of AIS lengths in hippocampus shows similar distribution in control and *db/db* mice at 5 weeks of age. Control, total 45 AISs from three mice; *db/db*, total 59 AISs from four mice. **(D)** Cumulative frequency distribution plots of AIS lengths in hippocampus of control and *db/db* mice at 10 weeks of age. Control without exercise, total 59 AISs from four mice; *db/db* mice without exercise, total 65 AISs from four mice; control with exercise, total 48 AISs from four mice; *db/db* mice with exercise, total 61 AISs from four mice. These four groups show similar pattern of distribution of AIS lengths, although the plot of *db/db* mice without exercise is shifted to the left. **(E,F)** Scatter plots of mean AIS length (y-axis) in relation to levels of blood glucose **(E)** or HbA1c **(F)** (x-axis) of individual mouse at 10 weeks of age.

In addition to the cerebral cortex, previous studies indicate that the hippocampus is involved in neuropsychiatric complications associated with type 2 diabetes in patients (Gold et al., 2007) and in *db/db* mice (Li et al., 2002). Therefore, we also investigated whether AIS was shortened in the hippocampus of the same animals used for prefrontal cortex analyses. Similar to the medial prefrontal cortex (Figures 1A, 4A), NeuN-positive neurons and AIS immunostaining appeared similar in the hippocampus CA1 area in male control and *db/db* mice without exercise (Figure 6A). We also examined expression of neuron, myelin, and AIS proteins in hippocampus in 10-week-old male control and *db/db* mice (both without exercise). Consistent with preserved hippocampal morphology (Figure 6A), and similar to the prefrontal cortex (Figures 4B,C), there were no differences in the protein levels of NeuN, MBP, or βIV spectrin (Figures 6B,C), as well as cleaved caspase 3 (Supplementary Figures S1B, S4). Finally, we analyzed AIS length in NeuN-positive neurons in the hippocampus. At 5 weeks of age, there was no difference in AIS length between control (31.03 ± 1.145 μm, *n* = 3 mice) and *db/db* (31.22 ± 0.12 μm, *n* = 4 mice) mice (*p* = 0.8546, unpaired *t*-test; Figures 7A,C). At 10 weeks, in the no exercise group, the AIS were significantly shorter in *db/db* (27.56 ± 1.47 μm, *n* = 4 mice) compared to control (32.81 ± 0.68 μm, *n* = 4 mice) mice (*p* = 0.0184, two-way ANOVA followed by Tukey’s; Figures 7B,D), similar to the AIS in medial prefrontal cortex (Figures 1, 3). No AIS shortening was observed in *db/db* mice after exercise treatment: AIS length was similar in exercised control (33.29 ± 1.15 μm, *n* = 4 mice) and exercised *db/db* (31.01 ± 0.65 μm, *n* = 4 mice) mice (*p* = 0.4449, two-way ANOVA followed by Tukey’s; Figures 7B,D). Furthermore, we found significant inverse correlation between AIS length and the levels of blood glucose (*r* = −0.6567, *p* = 0.0057, *n* = 16; Figure 7E) as well as HbA1c (*r* = −0.5155, *p* = 0.0410, *n* = 16; Figure 7F) in these mice. Taken together, our results suggest that the development of type 2 diabetes leads to AIS shortening in both medial prefrontal cortex and hippocampus, the brain regions that are implicated in development of cognitive impairment and psychiatric symptoms in patients with type 2 diabetes.

## Discussion

The current results are the first to show AIS shortening associated with type 2 diabetes, which is supported by three experimental results: (i) shortened AIS was observed in *db/db* mice at 10 weeks of age but not at 5 weeks; (ii) AIS length was inversely correlated with signs of type 2 diabetes, and (iii) AIS shortening was not observed after exercise treatment that improved glycemic indices. Despite striking similarity of molecular composition and their function, nodes of Ranvier were not affected, further highlighting the importance of AIS changes as a key axonal pathology during diabetic condition. Our findings may contribute to the understanding of mechanisms behind impaired cognitive function and mood previously reported in *db/db* mice (Li et al., 2002; Sharma et al., 2010; Dinel et al., 2011) and patients with type 2 diabetes (Stoeckel et al., 2016).

Impairment of cognitive function is becoming more frequently recognized as a major neuropsychiatric deficit that interferes with the daily life of patients with type 2 diabetes (for more detail, see review; Wrighten et al., 2009; Thabit et al., 2012; Stoeckel et al., 2016)). In the current study, we focused on the prefrontal cortex and hippocampus as potential sites of AIS alteration for several reasons. First, neuroanatomical abnormalities in the prefrontal cortex are observed using MRI of patients with type 2 diabetes (Kumar et al., 2008). Second, the prefrontal cortex is strongly associated with cognitive flexibility executive function (Birrell and Brown, 2000; Bissonette et al., 2013) and depression (Ballmaier et al., 2004). Both impaired cognitive flexibility (Thabit et al., 2012; Sadanand et al., 2016) and depression (Anderson et al., 2001; Doyle et al., 2014) are reported in patients with type 2 diabetes, in addition to the depression-like behavior reported in *db/db* mice (Sharma et al., 2010). Third, there are a number of reports describing hippocampus-mediated memory decline and associated brain changes in patients with type 2 diabetes (Gold et al., 2007) and *db/db* mice (Li et al., 2002; Stranahan et al., 2008; Chen et al., 2014; Infante-Garcia et al., 2017; Zheng et al., 2017). Our data show AIS shortening occurs in both medial prefrontal cortex and hippocampus in *db/db* mice at 10 weeks of age, during a period when blood glucose and HbA1c are elevated. AIS shortening occurred in the absence of changes in the morphological appearance of the medial prefrontal cortex or hippocampus, or in expression levels of neuronal and myelin proteins. Therefore, it is unlikely that AIS shortening is induced by neuronal loss (Ramos-Rodriguez et al., 2013) or disorganized myelin (Nam et al., 2017) observed in rodent models at more advanced stages of type 2 diabetes.

Previous studies suggest that type 2 diabetes alters synaptic plasticity in the hippocampus resulting in cognitive deficits in *db/db* mice (Li et al., 2002; Stranahan et al., 2008). Dendritic spines are the primary sites of excitatory synaptic inputs on pyramidal neurons, and the numbers of dendritic spines correlate with changes in cognitive function and mood (reviewed in von Bohlen Und Halbach, 2009; Kasai et al., 2010)). In *db/db* mice, dendritic spine density is decreased in dentate gyrus granule neurons in the hippocampus (Stranahan et al., 2009), hippocampal CA1 (Chen et al., 2014) and CA3 pyramidal neurons (Dhar et al., 2014), and layer II/III pyramidal neurons in prefrontal cortex (Chen et al., 2014). Reduced spine density may occur as early as postnatal day 28–32 in *db/db* mice (Dhar et al., 2014). Similar to the effects of exercise treatment in the current study (Figures 3, 7), voluntary running wheel activity attenuated reduction of dendritic spine density in hippocampus in *db/db* mice (Stranahan et al., 2009). Thus, the current understanding is that reduced synaptic inputs contribute to cognitive impairment during type 2 diabetes.

Emerging evidence indicates that activity-dependent functional plasticity of AIS structures plays a key role in maintaining neural circuit activity (reviewed in Yamada and Kuba, 2016; Jamann et al., 2018). For example, deprivation of auditory input to avian brainstem auditory neurons caused AIS elongation, which was associated with increased neuronal excitability, presumably as an adaptive response to compensate for the loss of auditory nerve activity (Kuba et al., 2010). Similarly, AIS elongation was observed after sensory deprivation in developing visual cortex (Gutzmann et al., 2014). This homeostatic plasticity is proposed to be a mechanism that maintains network excitability by modulating neuronal output in response to neuronal input changes. If this were true in type 2 diabetes condition, reduced dendritic spines (described above) would lead to decreased input and compensatory AIS lengthening and/or relocation. However, both AIS shortening (current study) and reduced dendritic spine density (Stranahan et al., 2009; Chen et al., 2014; Dhar et al., 2014) are reported in similarly aged *db/db* mice displaying cognitive and mood dysfunction (Li et al., 2002; Sharma et al., 2010). This suggests that AIS shortening is a pathological change caused by type 2 diabetes, rather than adaptive or compensatory change to maintain neural circuit activities.

What is the functional consequence of AIS shortening in diabetic brain? The 8%–16% decrease in AIS length reported herein is likely functionally relevant—a computational model showed that just a 4.5% decrease in AIS length reduces neuronal excitability (Baalman et al., 2013). Similarly, shortening of AIS in hippocampal neuron culture by 25% was associated with dampened neuronal excitability (Evans et al., 2015). Thus, AIS shortening due to diabetic conditions in *db/db* brains observed in this study might be a primary disease pathology that likely results in decreased neuronal excitability, further exacerbating the neuronal dysfunction caused by reduced dendritic spine density (see above). The idea of pathological AIS shortening in diabetes is further supported by previous reports associating AIS shortening with CNS pathology in rodent models of stroke (Hinman et al., 2013), mild traumatic brain injury (Baalman et al., 2013; Vascak et al., 2017), Alzheimer’s disease (Marin et al., 2016), or multiple sclerosis-related experimental autoimmune encephalitis model (Clark et al., 2016). Our results showing no difference in the expression levels of βIV spectrin, despite AIS shortening, are consistent with a previous study in a mild traumatic brain injury model showing AIS shortening without changes in the levels of AIS proteins (Baalman et al., 2013). Thus, our results strongly suggest that the modulation of neuronal output by AIS shortening is involved in the pathophysiology of neuropsychiatric complications during type 2 diabetes. Future studies could determine the contribution, if any, of AIS shortening to cognitive and mood impairment in *db/db* mice.

While our current results demonstrate that AIS is shortened in association with the development of type 2 diabetes, several important questions remain. For example, is the AIS modulated in the setting of type 1 diabetes? Similar to type 2 diabetes conditions discussed above, learning and memory deficits are also reported in patients with early-onset type 1 diabetes (Semenkovich et al., 2016a,b). The streptozotocin model of type 1 diabetes is associated with depression (Castillo-Gómez et al., 2015), learning and memory impairment (Biessels et al., 1996; Stranahan et al., 2008), reduced long term potentiation in the prefrontal cortex (Wu et al., 2017), and reduced dendritic spine density in hippocampus (Wang et al., 2014; Xiang et al., 2015) and prefrontal cortex (Wu et al., 2017). In addition, are all neurons equally affected by diabetes? Even though it is likely that AIS shortening occurs to a similar degree in all populations of neurons (Figure 1D), this is not conclusive, because detailed information for cell-type-specific AIS shortening is lacking. Previous studies show that neuronal cell-type is important in either diabetic brain complications or AIS morphology. Loss or changes of specific types of inhibitory neurons have been reported in type 2 diabetic Goto-Kakizaki rats (Larsson et al., 2016). AIS length is substantially heterogeneous in interneurons and pyramidal neurons (Höfflin et al., 2017), and the pattern of activity-dependent AIS changes is different between inhibitory interneurons and excitatory neurons (Chand et al., 2015). Cell-type-specific analyses of AIS changes could be an important next step to better understand pathophysiology of diabetic brain complications.

Finally, what is the molecular and cellular mechanism of AIS shortening in diabetic brain? Previous studies indicate that both homeostatic plasticity and pathological alterations of the AIS involve changes in intracellular calcium (Ca^2+^) levels and calcium-dependent enzymes. Pathological AIS alterations are mediated by calpain, a calcium-dependent cysteine protease, in models of stroke (Schafer et al., 2009), induced excitotoxicity (Del Puerto et al., 2015; Benned-Jensen et al., 2016), and multiple sclerosis (Clark et al., 2016). In addition, hyperphosphorylated tau (Hatch et al., 2017) or oxidative stress followed by calpain activation (Clark et al., 2017) induce pathological alterations of AIS. Calcium/calmodulin-dependent kinase II (CaMKII) may regulate excitability through its interaction with ankyrinG and βIV spectrin complex that anchor it to the AIS (Hund et al., 2010). Interestingly, a study looking at proteomic profile in *db/db* mice found altered expression of CaMKIIα, CaMKIIβ, CaMKIIδ and calcineurin subunit B type 1 both in hippocampus and frontal cortex (Ernst et al., 2013), and post-translational O-GlcNAcylation of CaMKII is increased in the diabetic brain (Erickson et al., 2013). Calcineurin, a calcium/calmodulin-dependent protein phosphatase, is responsible for both activity-dependent relocation (Evans et al., 2013) and shortening (Evans et al., 2015) of the AIS. Furthermore, AIS assembly, maintenance, and/or plasticity may also be regulated by cannabinoid receptors (Tapia et al., 2017), brain-derived neurotrophic factor and neurotrophin 3 (Guo et al., 2017), protein kinase CK2 (Bréchet et al., 2008; Hien et al., 2014; Xu and Cooper, 2015; Lezmy et al., 2017), Cdk5 (Trunova et al., 2011; Chand et al., 2015), microtubule cross-linking factor 1 (Satake et al., 2017), myosin II activity (Evans et al., 2017; Berger et al., 2018), or Rbfox splicing factors (Jacko et al., 2018). Future studies are required to identify the key mechanism of diabetes-related AIS changes.

In conclusion, this is the first study to show structural changes at the AIS associated with the development of type 2 diabetes. We report AIS shortening in two brain regions that are critical for appropriate cognitive function, affect, and learning and memory. In addition to the synaptic plasticity modulating *neuronal inputs*, the impairment of AIS plasticity regulating *neuronal outputs* might contribute to development of neuropsychiatric symptoms during type 2 diabetes. Thus, treatments aiming to restore AIS length might be a novel strategy to ameliorate cognitive and mood impairments in type 2 diabetes.

## Author Contributions

LY, KE and KS conceived and designed the research. LY, DD, and RG conducted the experiments and analyzed the data. LY and KS wrote the manuscript. DD and RG wrote sections of the manuscript; all authors contributed to manuscript revision, read and approved the submitted version.

## Conflict of Interest Statement

The authors declare that the research was conducted in the absence of any commercial or financial relationships that could be construed as a potential conflict of interest.
