# Supplementary material for: Type 2 Diabetes Leads to Axon Initial Segment Shortening in db/db Mice
Source: Front Cell Neurosci. 2018 Jun 8;12:146. doi: 10.3389/fncel.2018.00146 (PMC6002488; doi:10.3389/fncel.2018.00146)
Supplement: Supplementary file 1 [file Image_1.PDF]

Supplementary Material

Supplementary Figure S1.

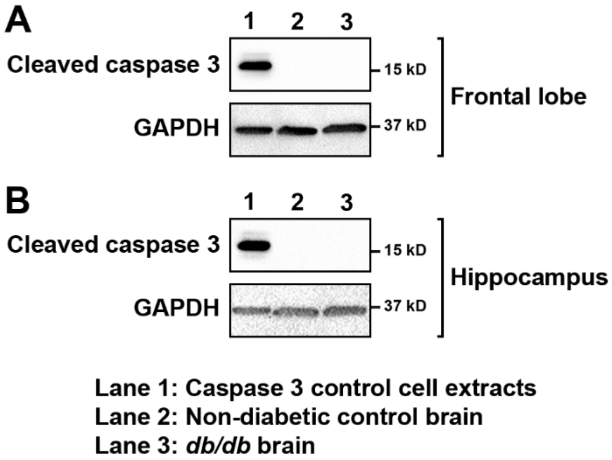

Supplementary Figure S1. Cleaved caspase 3 western blot.

Representative immunoblots of homogenized frontal brain **(A)** or hippocampus **(B)** from control and *db/db* mice at 10 weeks of age. Jurkat cells + Cytochrome c (Cell Signaling Technology Cat#9663) in lane 1 served as positive control for antibody. Uncropped images of the entire original immunoblots are shown in Supplementary Figure S4.

Supplementary Figure S2.

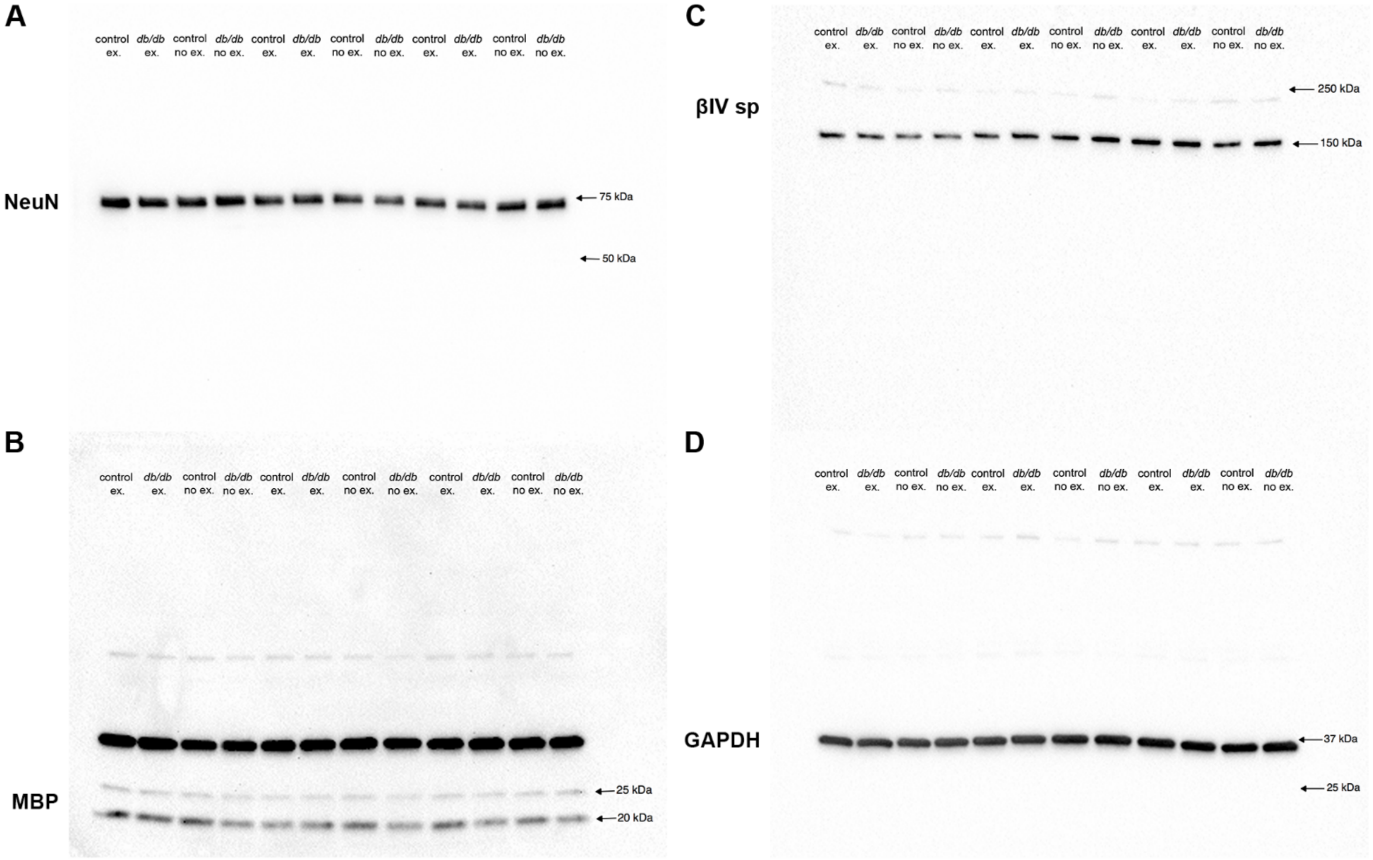

Supplementary Figure S2. Original immunoblots for Fig 4B.

Original full-size immunoblots of homogenized frontal brains, stained with antibody to NeuN **(A)**, MBP **(B)**, βIV spectrin **(C)**, and GAPDH **(D)**.

Supplementary Figure S3.

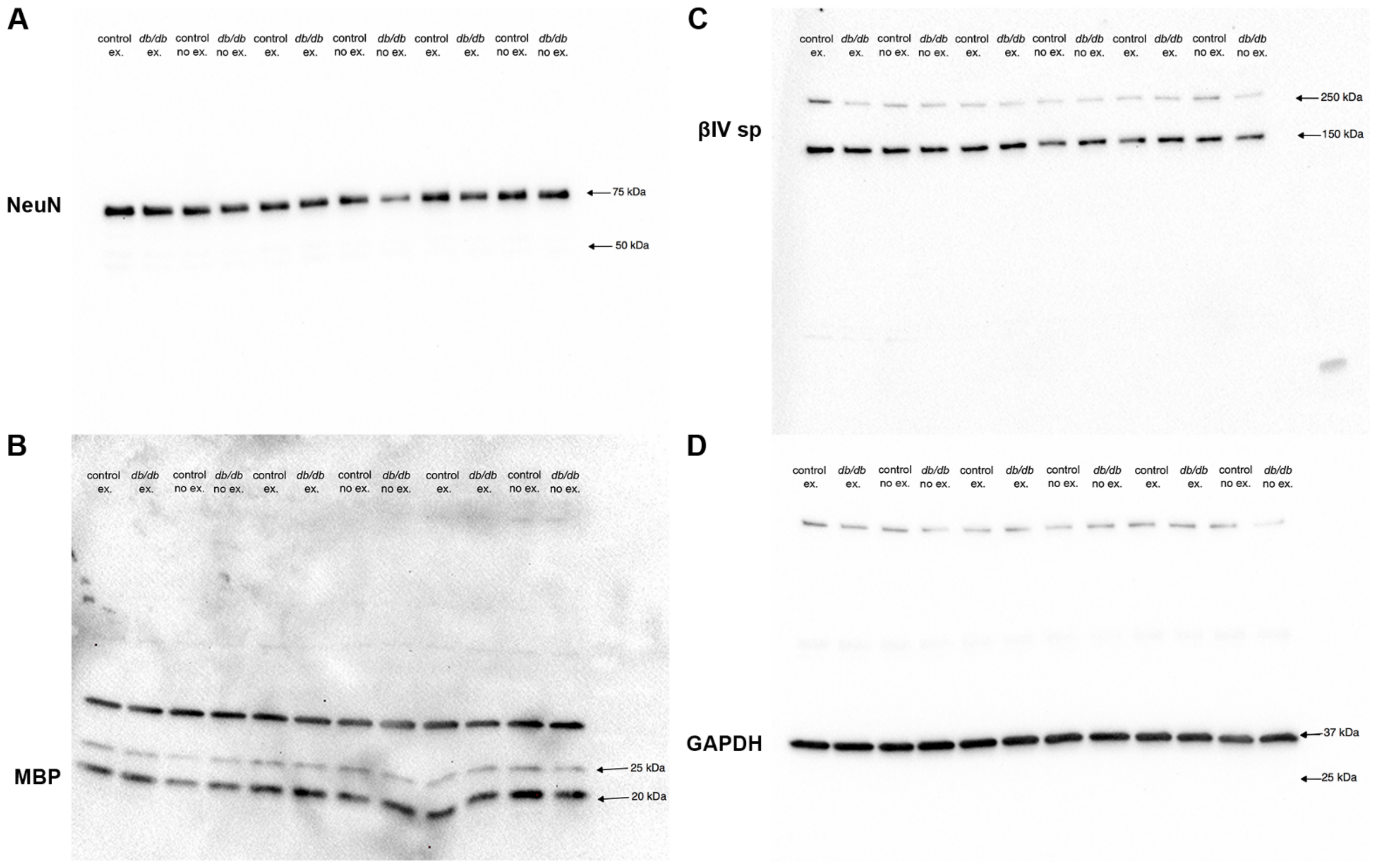

Supplementary Figure S3. Original immunoblots for Fig 6B.

Original full-size immunoblots of homogenized hippocampi, stained with antibody to NeuN **(A)**, MBP **(B)**, βIV spectrin **(C)**, and GAPDH **(D)**.

Supplementary Figure S4.

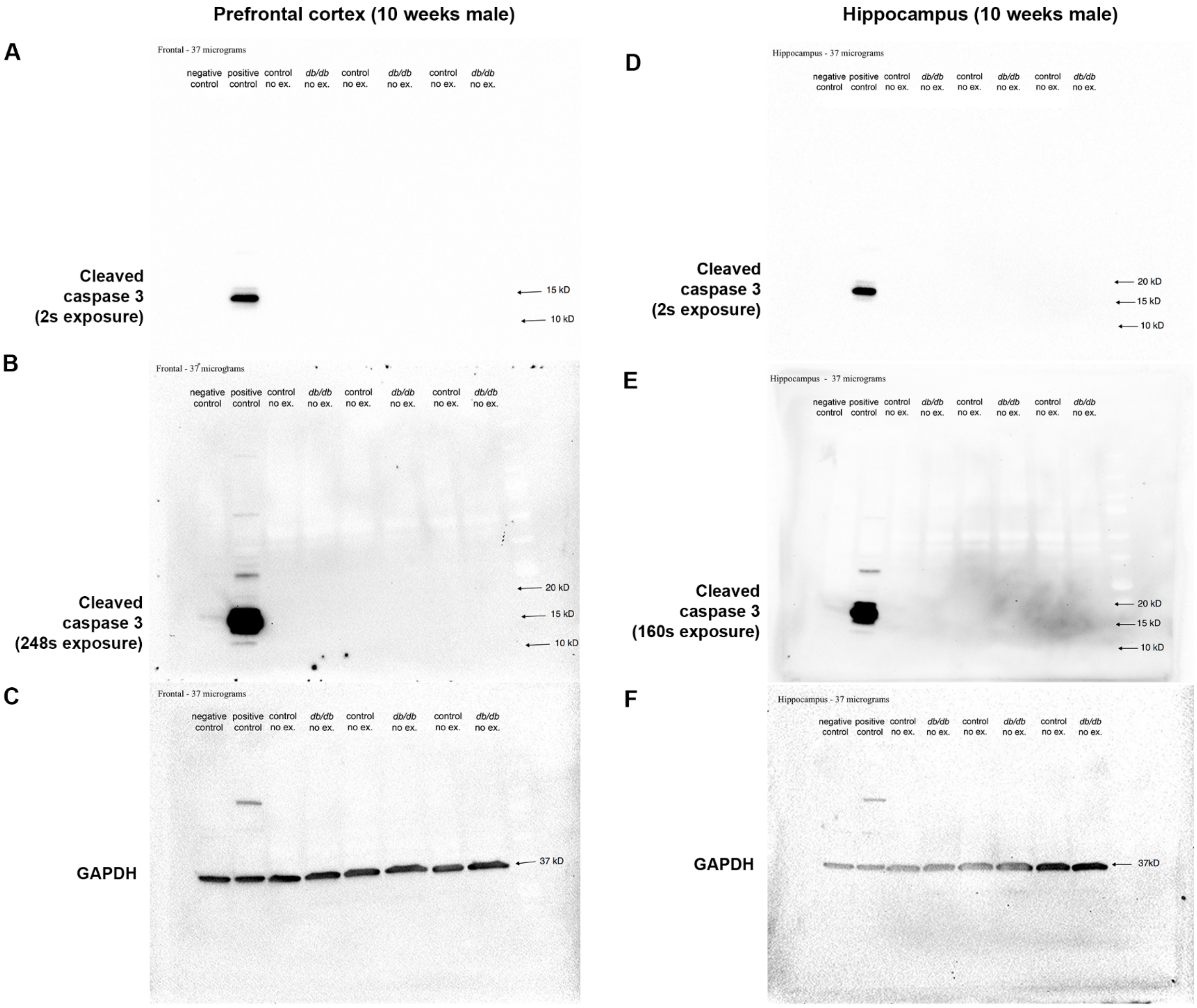

Supplementary Figure S4. Original immunoblots for Supplementary Figure S1.

(A-C) Original full-size immunoblot of homogenized frontal lobes, stained with antibody to cleaved caspase 3, with short exposure time **(A)** or long exposure time **(B)**; and stained with antibody to GAPDH **(C)**. Untreated Jurkat (negative control) and Jurkat cells + Cytochrome c (positive control) were used as controls (Cell Signaling Technology Cat#9663).

(D-F) Original full-size immunoblot of homogenized hippocampi, stained with antibody to cleaved caspase 3, with short exposure time **(D)** or long exposure time **(E)**; and stained with antibody to GAPDH **(F)**.
